# Status of cardiac arrhythmia services in Africa in 2018: a PASCAR Sudden Cardiac Death Task Force report

**DOI:** 10.5830/CVJA-2018-027

**Published:** 2018

**Authors:** Talle MA, Bonny A, Scholtz W, Nel G, Chin A, Karaye KM, Sani MU, Anzouan-Kacou JB, Damasceno A, Lubenga YR, Mayosi BM

**Affiliations:** Cardiology Unit, Department of Medicine, University of Maiduguri Teaching Hospital, Maiduguri, Nigeria; University of Douala, Cameroon Cardiovascular Research Network, Douala, Cameroon; Hopital Forcilles, Ferolles–Attilly, France; Pan-Africa Society of Cardiology (PASCAR); Pan-Africa Society of Cardiology (PASCAR); Cardiac Clinic, Department of Medicine, Groote Schuur Hospital and University of Cape Town, Cape Town, South Africa; Bayero University and Aminu Kano Teaching Hospital, Department of Cardiology, Kano, Nigeria; Bayero University and Aminu Kano Teaching Hospital, Department of Cardiology, Kano, Nigeria; Felix Houphouet Boigny University, Abidjan, Ivory Coast; Cardiology Institute of Abidjan, Ivory Coast; Faculty of Medicine, Eduardo Mondlane University, Maputo, Mozambique; Department of Cardiology, University Hospital of Kinshasa, Democratic Republic of Congo; Department of Medicine, Faculty of Health Sciences, University of Cape Town and Groote Schuur Hospital, Cape Town, South Africa

**Keywords:** cardiovascular diseases, cardiac arrhythmias, Africa, management, services

## Abstract

**Background:**

There is limited information on the availability of health services to treat cardiac arrhythmias in Africa.

**Methods:**

The Pan–African Society of Cardiology (PASCAR) Sudden Cardiac Death Task Force conducted a survey of the burden of cardiac arrhythmias and related services over two months (15 October to 15 December) in 2017. An electronic questionnaire was completed by general cardiologists and electrophysiologists working in African countries. The questionnaire focused on availability of human resources, diagnostic tools and treatment modalities in each country.

**Results:**

We received responses from physicians in 33 out of 55 (60%) African countries. Limited use of basic cardiovascular drugs such as anti–arrhythmics and anticoagulants prevails. Non–vitamin K–dependent oral anticoagulants (NOACs) are not widely used on the continent, even in North Africa. Six (18%) of the sub–Saharan African (SSA) countries do not have a registered cardiologist and about one–third do not have pacemaker services. The median pacemaker implantation rate was 2.66 per million population per country, which is 200–fold lower than in Europe. The density of pacemaker facilities and operators in Africa is quite low, with a median of 0.14 (0.03–6.36) centres and 0.10 (0.05–9.49) operators per million population. Less than half of the African countries have a functional catheter laboratory with only South Africa providing the full complement of services for cardiac arrhythmia in SSA. Overall, countries in North Africa have better coverage, leaving more than 110 million people in SSA without access to effective basic treatment for cardiac conduction disturbances.

**Conclusion:**

The lack of diagnostic and treatment services for cardiac arrhythmias is a common scenario in the majority of SSA countries, resulting in sub–optimal care and a subsequent high burden of premature cardiac death. There is a need to improve the standard of care by providing essential services such as cardiac pacemaker implantation.

Cardiovascular diseases (CVD) including cardiac arrhythmias are a major public health problem in low– and middle–income countries (LMICs) to which all sub–Saharan African (SSA) countries belong.^1^,^2^ Lack of adequate data on the real burden of cardiac arrhythmias and the need for expensive equipment and drugs poses a great impediment to the management of patients with arrythmogenic diseases in SSA.

Accurate diagnosis and treatment of cardiac arrhythmias require monitoring of electrical activity of the heart, drug challenges to unmask paroxysmal/concealed arrhythmias, and non–invasive and invasive imaging techniques, which are not affordable in many African countries. In addition, there are disparities in cardiovascular healthcare between countries, which are not properly documented. These disparities need to be established for developing south–south collaboration between more developed countries and those with a lack of facilities.

The first studies of pacemaker and implantable cardioverter defibrillator implantation rates in SSA were conducted about 20 years ago.^3^–^5^ These studies, together with a recent evaluation of access and use of cardiac implantable electronic devices (CIED) and catheter ablation procedures show very low levels of use and access to CIEDs in SSA. The Pan–African Society of Cardiology (PASCAR) Sudden Cardiac Death Task Force has conducted the first pan–African survey on human resources, diagnostic tools and treatment for cardiac arrhythmias across African countries.

## Methods

We conducted a survey across African countries using an electronic questionnaire (Table 1) between 15 October and 15 December 2017. This was completed by general cardiologists and electrophysiologists from the PASCAR community. The questionnaire focused on availability of human ressources, diagnostic work up and treatment in each country. Blinded multiple responders per country were requested as much as possible. In case of significant disparities in the information from multiple responders, they were invited to revise their responses. For countries in SSA that do not have cardiac physicians, information was obtained from official authorities such as the embassy of the country in question.

**Table 1:** Questionnaire administered for the survey of arrhythmia services in Africa

1. In which country are you currently practicing medicine?	
2. In which centre are you performing electrophysiology and/or pacing? Select all applicable options.	
a. Public non-teaching hospital	b. Teaching hospital
c. Private sector	
3. In how many centres in your country are patients able to receive	
a. Pacemaker implantation	
b. CRT (cardiac resynchronisation therapy)	
c. ICD (implantable cardioverter–defibrillator)	
d. Catheter ablation procedures?	
4. What is the percentage of public hospitals in your country that supply electrophysiology and/or pacing?	
5. Where did you receive your training in electrophysiological procedures and/or pacing?	
a. Current country	b. Another African country (please specify)
c. Asia	d. Europe
e. America	
6. Have you travelled abroad for a short-term fellowship in electrophysiological procedures and/or pacing? If yes, where?	
a. No	b. Another African country (please specify)
c. Asia	d. Europe
e. America	f. Australasia
7. Which of the following procedures you are able to perform? Select all applicable options.	
a. Pacemaker	
b. CRT (cardiac resynchronisation therapy)	
c. ICD (implantable cardioverter–defibrillator)	
d. Catheter ablations	
e. None
8. How many physicians in your country are able to	
a. Implant pacemakers	b. Implant ICD
c. Implant CRT	d. Perform ablation procedures?
9. Which of the following diagnostic drugs are available in your country? Please select all applicable options	
a. Ajmaline iv	b. Flecanide iv
c. Other, please specify	
10. Which of the following anti-arrhythmia drugs are available in your country? Please select all applicable options	
a. Amiodarone iv	b. Xylocaine iv
c. Beta-blockers iv	d. Digoxine iv
e. Procainamide	f. Flecainide
g. Hydroquinidine	
11. Which of the following are used in the treatment of atrial fibrillation/flutter in your country? Please select all applicable options	
a. Aspirin	b. Clopdiogrel
c. VKA (Vitamin K antagonist)	d. Apixaban
e. Rivaroxaban	f. Dabigatran
g. Electrical cardioversion	h. Flutter ablation
i. AV node ablation	j. AF ablation (pulmonary vein isolation)
12. Which of the following diagnostic tools are used in your country? Please select all applicable options	
a. 12-lead ECG	b. Signal-averaged ECG
c. Holter ECG	d. 2D echo
e. Tilt-table testing	f. Exercise testing
i. Right ventricle angiography	j. Cardiac CT scan
k. Cardiac MRI	
13. Which of the following invasive therapies are used in your country? Please select all applicable options	
a. Pacemaker implantation	b. CRT implantation
c. ICD implantation	d. Flutter ablation
e. AV nodal re-entry ablation	f. Accessory pathway ablation	g. AF ablation (pulmonary vein isolation)
h. Complex ablation requiring 3D mapping (ventricular tachycardia, PVCs, atrial tachycardia)	
14. Please provide contact details (voluntary) to allow us to keep you abreast of Developments	
a. Title, name and surname	b. Institution name
c. E-mail address	d. Mobile number

## Results

Of the 55 African countries, data from 33 countries were available. In 19 (63%) of the countries, data were provided from at least two responders. 

## Human resources

**Fig. 1:**
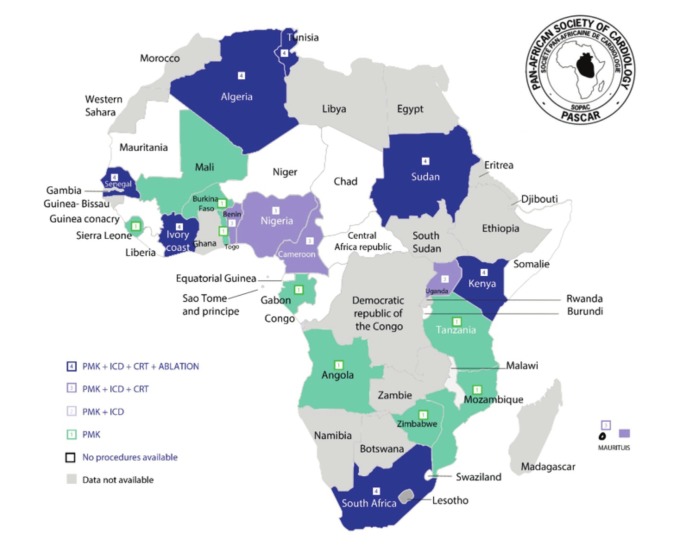
Availability of various cardiac arrhythmia services across the African continent. PMK = pacemaker; ICD = implantable cardioverter defibrillator; CRT = cardiac resynchronisation therapy. Ablation includes radiofrequency ablation for atrial flutter and junctional tachycardia (simple ablation) as well as catherter ablation for atrial fibrillation (complex ablation).

To the best of our knowledge, six (18%) countries did not have a registered cardiologist in 2017. These were Central African Republic, Equatorial Guinea, Liberia, Malawi, Somalia and Swaziland. In addition, 11 (33.3%) countries had no trained physicians capable of performing pacemaker implantations (Fig. 1). Fellowship training courses in cardiac pacing for physicians and technologists to provide the required expertise were available in all North African countries, in contrast to SSA countries where this exists in South Africa only.

**Fig. 2:**
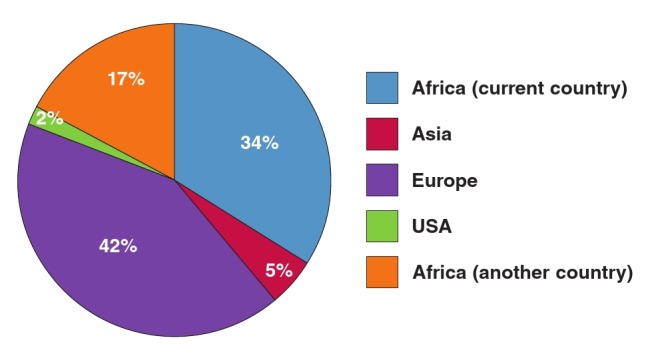
Centres where primary training in cardiac pacing was obtained by cardiologists practicing in Africa. More than 50% of the cardiologists were trained in Africa.

**Fig. 3:**
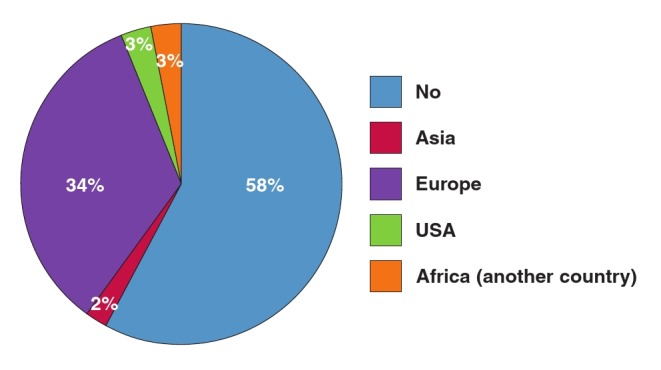
Centres where short–term fellowship in cardiac pacing was obtained by cardiologists practicing in Africa; 58% have not had formal fellowship training in cardiac pacing.

As shown in Fig. 2, most operators obtained their baseline skills in Europe, followed by their own country or another African country, and the minority in Asia or North America. More importantly, almost 60% of these experts did not receive any postgraduate training overseas, and only one–third benefited from a fellowship programme in Europe (Fig. 3). The University of Cape Town has trained three fellows in cardiac pacing from Tanzania, Sierra Leone and Kenya as part of the PASCAR fellowship in cardiac pacing, and a similar training programme has been launched at the University of Gaston Berger, Saint Louis, Senegal.

## Diagnostic facilities and challenges

Electrocardiography (ECG): as shown in Table 2, ECG was widely available in all African countries, although mainly in secondary and tertiary health facilities. Signal–averaged ECG (SA–ECG), which detects arrhythmogenic late potentials for the diagnosis of arrhythmogenic right ventricular cardiomyopathy/ dysplasia (ARVC/D) was available only in South Africa and in Maghreb.

Ambulatory ECG monitoring (24–hour Holter–ECG) and exercise treadmill testing were not routinely performed in many countries. These tests were available in only 76 and 61% of the countries, respectively, and they were very expensive. For instance, in Cameroon, they cost approximately US$180, about 2.7–fold higher than the minimum monthly wage.

**Table 2:** Routine diagnostic techniques available in the various African countries

*Countries*	*ECG*	*SA-ECG*	*Holter ECG*	*2D echo*	*Tilt-table testing*	*Exercise testing*
South Africa	X	X	X	X	X	X
Sudan	X	X	X	X	X	X
Algeria	X	x	X	X	X	X
Tunisia	X	X	X	X		X
Senegal	X		X	X	X	X
Côte d’ivoire	X		X	X	X	X
Kenya	X		X	X	X	X
Nigeria	X		X	X		X
Mauritius	X		X	X		X
Cameroon	X		X	X		X
Angola	X		X	X		X
Tanzania	X		X	X		X
Mozambique	X		X	X		X
Sierra Leone	X		X	X		X
Burkina Faso	X		X	X		X
Zimbabwe	X		X	X		X
Burundi	X		X	X		X
Uganda	X		X	X		X
Benin	X		X	X		X
Gabon	X		X	X		X
Chad	X		X	X		
Congo Republic	X		X	X		
Mali	X		X	X		
Togo	X		X	X		
Mauritania	X		X	X		
Equatorial Guinea	X			X		
Guinea Conakry	X			X		
Somalia	X			X		
Niger	X			X		
Malawi	X					
Swaziland	X					
Liberia	X					
Central Africa Republic	X					

SA-ECG and tilt-table test are available in a minority. ECG = electrocardiography; SA-ECG = signal-averaged electrocardiography; 2D echo = two-dimentional echocardiography.

Other diagnostic techniques: echocardiography was the most commonly used imaging technique to rule out or confirm structural heart diseases. Although widely available in Africa (Table 2), its use was limited to tertiary centres in larger cities. Tilt–testing to rule out vaso–vagal syncope was available in only six (18.2%) countries (Table 2).

Electrophysiological procedures (EP) for the diagnosis of paroxysmal advanced heart blocks or tachyarrhythmias were routinely performed only in countries from North Africa, Kenya, Senegal and South Africa. Other SSA countries with implantation activity were able to supply pacemaker implantations to only patients with overt conduction system disturbances, excluding the remaining population with unexplained syncope. A catheter laboratory was not available in 19 (57.6%) countries (Fig. 1).

**Fig. 4:**
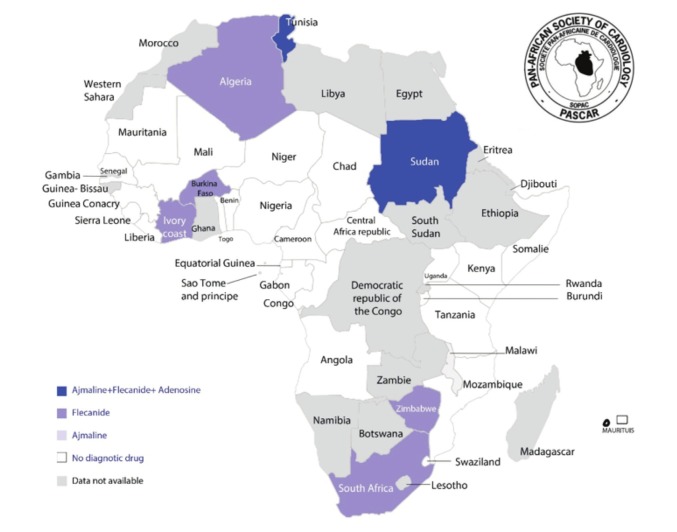
Availability of drugs used for unmasking covert cardiac arrhythmias.

Drug challenge aims to unmask silent phenotypes of inheritable arrhythmogenic diseases such as Brugada syndrome. However, this test was largely unavailable in many countries of SSA, with anecdotal reports on ajmaline or flecanide use in some countries (Fig. 4).

## Treatments

Anti–arrhythmia drugs: as shown in the Table 3, digoxin and amiodarone were the most commonly used medications. Vaughan William’s class I anti–arrhythmic drugs were in short supply, apart from flecainide, which was present in about 50% of the countries. In general, parenteral medications were less available, making the management of life–threatening arrhythmias quite challenging.

**Table 3:** Availability of various anti-arrhythmic drugs in various African countries

*Country*	*Digoxine*	*Amiodarone*	*Beta-blockers*	*Flecainide*	*Xylocaïne iv*	*Procainamide iv*	*Hydroquinidine*
South Africa	X	X	X	X	X	X	
Tunisia	X	X	X	X			X
Sudan	X	X	X	X	X		
Côte d’ivoire	X	X	X	X	X		
Algeria	X	X	X	X	X		
Burkina Faso	X	X	X	X	X		
Kenya	X	X	X	X	X		
Gabon	X	X	X	X			
Uganda	X	X	X	X			
Tanzania	X	X	X				
Sierra Leone	X	X	X				
Angola	X	X	X		X		
Nigeria	X	X	X		X		
Senegal	X	X	X		X		X
Niger	X	X	X				
Zimbabwe	X	X		X	X		
Mauritius	X	X		X			
Mozambique	X	X			X		
Burundi	X	X					
Mauritania	X	X					
Benin	X	X					
Cameroon	X			X			
Guinea Conakry	X						
Congo Republic	X						
Mali	X						
Togo	X						
Liberia	X						
Chad	X						
Equatorial Guinea							
Somalia							
Malawi							
Swaziland							
Central Africa Republic							

Procainamide and hydroquinidine are largely unavailable; iv = intravenous.

Anticoagulants: the increasing burden of atrial fibrillation (Afib) in Africa underscores the need for proper diagnosis and management in order to better prevent stroke and heart failure. Vitamin K antagonists (VKAs) were widely available in Africa (Table 4) but their optimal use is challenging. Two–thirds of the REMEDY Afib study patients with a CHA2DS2 –VASC score > 1 were on oral anti–coagulation but only 27.4% had INR in the therapeutic range.^5^

**Table 4:** Treatments available for atrial fibrillation and atrial flutter

*Country*	*Aspirin*	*VKA*	*Apixaban*	*Rivaroxaban*	*Dabigatran*	*Electrical Cardioversion*	*Flutter ablation*	*AV node ablation*	*AF ablation (pulmonary vein isolation)*
Tunisia	X	X				X	X	X	X
South Africa	X	X	X	X	X	X	X	X	X
Sudan	X	X	X	X	X	X			
Kenya	X	X		X	X	x	X	X	
Gabon	X	X		X	X	X			
Sierra Leone	X	X		X	X	X			
Nigeria	X	X		X	X	X			
Mauritius	X	X		X	X				
Algeria	X	X		X		X	X	X	x
Senegal	X	X		X		X	X	X	
Côte d’ivoire	X	X		X		X			
Tanzania	X	X		X		X			
Angola	X	X		X		X			
Zimbabwe	X	X		X					
Cameroon	X	X		X					
Uganda	X	X				X			
Niger	X	X				X			
Mozambique	X	X				X			
Burkina Faso	X	X				X			
Chad	X	x							
Guinea Conakry	X	x							
Congo Republic	X	x							
Mali	X	x							
Togo	X	X							
Burundi	X	X							
Mauritania	X	X							
Benin	X	x							
Central Africa Republic	X								
Equatorial Guinea	X								
Somalia	X								
Malawi	X								
Swaziland	X								
Liberia	X								

Ablation (simple and complex) remains largely unavailable in the region. VKA = Vitamin K antagonist; AV = atrioventricular; AF = atrial fibrillation.

Non–vitamin K–dependent oral anticoagulants (NOACs) were not available in the majority of countries, including North– African countries such as Tunisia (Table 4).

Invasive treatment: considerable heterogeneity in the access to invasive arrhythmia treatment was observed across Africa (Fig. 1). About one–third of the PASCAR countries did not perform pacemaker implantations: Burundi, Central African Republic, Chad, Equatorial Guinea, Guinea Conakry, Liberia, Malawi, Niger, Republic of Congo, Sao Tome et Principe, Swaziland and Somalia. In 2014, the median pacemaker implantation rate was 2.66 per million population per country.^7^ The 2017 PASCAR survey showed that the density of pacemaker facilities and operators in SSA was quite low, with a median of 0.14 centres per million population and 0.10 operators per million population.^7^ Implantable cardioverter–defibrillator (ICD) and cardiac resynchronisation therapy (CRT) were performed in 11/33 (33.3%) and 10/33 (30%) of the countries respectively.^7^

Electrophysiological studies and ablation techniques were unavailable in all SSA areas, apart from South Africa. Here complex ablations requiring three–dimensional mapping were routinely carried out, as in countries of the Maghreb (Table 5).

**Table 5:** Electrophysiological procedures including complex catheter ablations

*Country*	*PMK*	*CRT*	*ICD*	*Flutter ablation*	*AV nodal re-entry Ablation*	*Accessory pathway Ablation*	*AF ablation*	*Complex ablation requiring 3D mapping*
South Africa	X	X	X	X	X	X	X	X
Algeria	X	x	X	X	X	X	X	x
Kenya	X	X	X	X	X	X	X	
Tunisia	X	x	X	X	X	X		x
Senegal	X	X	X	X	X	X		
Sudan	X	X	x					
Côte d’ivoire	x	x	x					
Nigeria	x	x	X					
Mauritius	x	x	X					
Uganda	X	X	x					
Cameroon	X		x					
Benin	X		X					
Tanzania	x							
Mozambique	x							
Sierra Leone	x							
Angola	x							
Burkina Faso	x							
Zimbabwe	x							
Mali	x							
Togo	x							
Mauritania	x							
Gabon	x
Chad
Somalia								
Malawi								
Swaziland								
Liberia								
Burundi								
Central Africa Republic								
Equatorial Guinea								
Guinea Conakry								
Congo Republic								
Niger								

Marked variation in cost (up to 1 000–fold) was observed across countries, with an inverse correlation between implant rates and the procedural fees standardised to the gross domestic product (GDP) per capita.^7^ Poverty, lack of facilities/equipment, prohibitive costs of procedures, paucity of trained health professionnals, and non–existent fellowship programmes were the main drivers of under–utilisation of interventional cardiac arrhythmia care.

## Discussion

The paradigm shift in the epidemiology of disease burden in Africa towards the predominance of non–communicable diseases (NCDs) emphasises the need for appropriate health policies to address the changing pattern of diseases. The steady increase in the incidence of heart diseases and their risk factors, such as hypertension, ischaemic heart disease, diabetes and heart failure mechanistically impact significantly on the burden of cardiac arrhythmogenic disorders.^8^–^11^ Therefore, health policies in Africa should be aligned towards better management of cardiac arrhythmias.

Unfortunately, the current situation in many African countries is very worrisome. Our results show that there was a significant heterogeneity in both access to care and use of CIEDs and EP procedures across the African continent. In fact, the only SSA country with the full armamentarium of arrhythmia services was South Africa. This illustrates the magnitude of the challenge facing many African countries as they set out to improve and potentially expand cardiac arrhythmia care.

Given the high incidence of thromboembolic events among patients with rheumatic heart disease, the use of anticoagulation for Afib and INR monitoring need to be especially encouraged. So is the use of proven and relatively simple therapeutic options such as beta–blockers, amiodarone and class Ic antiarrhythmic drugs, as well as external electrical cardioversion for haemodynamically compromised cardiac arrhythmias, which require neither specific facilities nor technical expertise. This is the starting point for the treatment of tachyarrhythmias in those areas where resources are scarce. On the other hand, digoxin, which is the first–line agent for rate control of Afib in most SSA countries (Table 3), should be used cautiously, particularly in the setting of SSA where facilities for monitoring serum digoxin concentration are particularly lacking.^12^–^14^

There is a clear need to promote contemporary cardiac arrhythmia care in Africa and to address the disparity that exists between different regions and countries. This will be a huge and multi–faceted challenge. In Europe, the White Book data have been used successfully to raise awareness about inequalities in the treatment of arrhythmias, not only within the cardiology community but also among healthcare administrators, policymakers and other stakeholders.^15^ Similar steps now also need to be taken in Africa, using data from the first PASCAR arrhythmia report and the results of this survey, and focusing first on developing widespread basic arrhythmia services. 7

Given the disparities in care already in place, it is obvious that the requirements for improvement in various regions will differ substantially. One might also question how realistic it is to promote arrhythmia care in those areas where the healthcare infrastructure is weak, and even primary care is poorly organised and underfunded.

Ideally, basic arrhythmia services would include non–invasive diagnostic work up, intravenous and oral anti–arrhythmia drugs (including parenteral ones), anticoagulants (including NOACs), procedures such as pacemaker implantations, ICDs for secondary prevention, CRT and simple radiofrequency catheter ablations. Re–use of cardiac devices should be promoted to a greater extent, to include those unable to afford new devices.^16^ This will require significant investment in facilities and training of physicians in some areas.

For this reason, the development of arrhythmia services could initially be congregated in a few selected centres in Africa as centres of excellence, which could eventually also function as training sites for arrhythmia specialists. In this regard, countries of North Africa, Kenya, Senegal and South Africa are well equiped to drive such a south–south cooperation. In addition, telemedicine and e–cardiology, including ECG monitoring, will help to diagnose cardiac arrhythmias in patients living in poorresource settings lacking healthcare professionals.

## Study limitations

The data of the survey highlighting availability of facilities and treatments were mainly obtained on a declarative basis. Also, some countries in SSA had multiple responders while some had none or only one responder. These may have resulted in incomplete data. However, the data still provide an insight into the availability of cardiac arrhythmia services in Africa, which could be used for advocacy and planning. 

## Conclusion

This new pan–African survey on managing arrhythmias in Africa describes the current status and challenges of managing cardiac arrhythmias in different geographical regions of Africa. There are also huge disparities in diagnostic and treatment facilites in Africa. The increasing burden of cardiac arrhythmias and premature cardiac death calls for better understanding of cardiac arrhythmias, promoting awareness of the importance of arrhythmogenic cardiac disorders in the spectrum of tropical cardiac diseases, and improved cardiac arrhythmia services in Africa.

## References

[1] Mensah GA, Sampson UK, Roth GA (2015). Mortality from cardiovascular diseases in sub–Saharan Africa, 1990–2013: a systematic analysis of data from the Global Burden of Disease Study 2013. Cardiovasc J Africa.

[2] Bestawros M (2017). Electrophysiology in the developing world. Challenges and opportunities. Cardiol Clin.

[3] Mayosi BM, Scott–Millar R (1988). The 1995 survey of cardiac pacing in South Africa. S Afr Med J.

[4] Mayosi BM, Scott–Millar RN (2000). Permanent cardiac pacing in Africa. East Afr Med J.

[5] Zühlke L, Engel ME, Karthikeyan G (2014). Characteristics, complications, and gaps in evidence–based interventions in rheumatic heart disease: the Global Rheumatic Heart Disease Registry (the REMEDY study). Eur Heart J.

[6] Bonny A, Ngantcha M, Jeilan M, Okello E, Kaviraj B, Talle MA Statistics on the use of cardiac electronic devices and interventional electrophysiological procedures in Africa from 2011 to 2016: report of the Pan–African Society of Cardiology (PASCAR) Cardiac Arrhythmias and Pacing Task Forces. Europace. 2017; Dec 21. doi: 10.1093/europace/ eux353. [Epub ahead of print]..

[7] Sliwa K, Wilkinson D, Hansen C (2008). Spectrum of heart disease and risk factors in a black urban population in South Africa (the Heart of Soweto Study): a cohort study. Lancet.

[8] Tefera YG, Abegaz TM, Abebe TB (2017). The changing trend of cardiovascular disease and its clinical characteristics in Ethiopia: hospitalbased observational study. Vasc Health Risk Manag.

[9] Carlson S, Duber HC, Achan J, Stergachis A, Wollum A, Bukhman G (2017). Capacity for diagnosis and treatment of heart failure in sub– Saharan Africa. Heart.

[10] Bloomfield GS, Barasa FA, Doll JA (2013). Heart failure in sub–Saharan Africa. Curr Cardiol Rev.

[11] Ouyang AJ, Lv YN, Zhong HL, Wen JH, Wei XH, Peng HW (2015). Meta–analysis of digoxin use and risk of mortality in patients with atrial fibrillation. Am J Cardiol.

[12] Van Veldhuisen DJ, van Gelder IC, Ahmed A, Gheorghiade G (2013). Digoxin for patients with atrial fibrillation and heart failure : paradise lost or not?. Eur Heart J.

[13] Lopes RD, Rordorf R, de Ferrari GM (2018). Digoxin and mortality in patients with atrial fibrillation. J Am Coll of Cardiol.

[14] Raatikainen MJ, Arnar DO, Merkely B, Nielsen JC, Hindricks G, Heidbuchel H (2017). A decade of information on the use of cardiac implantable electronic devices and interventional electrophysiological procedures in the European Society of Cardiology countries: 2017 report from the European Heart Rhythm Association. Europace.

[15] Sani MU, Mayosi BM (2017). The Pacemaker and ICD Reuse Programme of the Pan–African Society of Cardiology. Heart.

